# Preventing Slips and Falls through Leisure-Time Physical Activity: Findings from a Study of Limited-Service Restaurants

**DOI:** 10.1371/journal.pone.0110248

**Published:** 2014-10-16

**Authors:** Alberto J. Caban-Martinez, Theodore K. Courtney, Wen-Ruey Chang, David A. Lombardi, Yueng-Hsiang Huang, Melanye J. Brennan, Melissa J. Perry, Jeffrey N. Katz, Santosh K. Verma

**Affiliations:** 1 Center for Injury Epidemiology, Liberty Mutual Research Institute for Safety, Hopkinton, Massachusetts, United States of America; 2 Environmental and Occupational Medicine and Epidemiology Program, Department of Environmental Health, Harvard School of Public Health, Boston, Massachusetts, United States of America; 3 Orthopaedic and Arthritis Center for Outcomes Research, Department of Orthopedic Surgery, Brigham and Women's Hospital, Boston, Massachusetts, United States of America, and Harvard Medical School, Boston, Massachusetts, United States of America; 4 Center for Physical Ergonomics, Liberty Mutual Research Institute for Safety, Hopkinton, Massachusetts, United States of America; 5 Center for Behavioral Sciences, Liberty Mutual Research Institute for Safety, Hopkinton, Massachusetts, United States of America; 6 Department of Environmental and Occupational Health, The George Washington University, Washington, D.C., United States of America; 7 Department of Family Medicine and Community Health, University of Massachusetts Medical School, Worcester, Massachusetts, United States of America; University of Rome, Italy

## Abstract

**Background/Objective:**

Physical activity has been shown to be beneficial at improving health in some medical conditions and in preventing injury. Epidemiologic studies suggest that physical activity is one factor associated with a decreased risk for slips and falls in the older (≥65 years) adult population. While the risk of slips and falls is generally lower in younger than in older adults; little is known of the relative contribution of physical activity in preventing slips and falls in younger adults. We examined whether engagement in leisure-time physical activity (LTPA) was protective of slips and falls among a younger/middle-aged (≤50 years old) working population.

**Methods:**

475 workers from 36 limited-service restaurants in six states in the U.S. were recruited to participate in a prospective cohort study of workplace slipping. Information on LTPA was collected at the time of enrollment. Participants reported their slip experience and work hours weekly for up to 12 weeks. We investigated the association between the rate of slipping and the rate of major slipping (i.e., slips that resulted in a fall and/or injury) and LTPA for workers 50 years of age and younger (n = 433, range 18–50 years old) using a multivariable negative binomial generalized estimating equation model.

**Results:**

The rate of major slips among workers who engaged in moderate (Adjusted Rate Ratio (RR)  = 0.65; 95% Confidence Interval (CI)  =  [0.18–2.44]) and vigorous (RR = 0.64; 95%CI  =  [0.18–2.26]) LTPA, while non-significant, were approximately one-third lower than the rate of major slips among less active workers.

**Conclusion:**

While not statistically significant, the results suggest a potential association between engagement in moderate and vigorous LTPA and the rate of major slips in younger adults. Additional studies that examine the role of occupational and non-occupational physical activity on the risk of slips, trips and falls among younger and middle aged adults appear warranted.

## Introduction

Risk factors for slips and falls among older adults (65 and older) have been well characterized [1]–[5]; however, less is known regarding factors that may predispose younger/middle-aged adults (≤50 years old) to slips and falls. Engagement in higher levels of physical activity is one factor that has been reported to be associated with a decreased risk for falling in the older adult population [6]–[8]. For example, regular physical activity among the older adult population has been shown to maintain mobility, physical functioning, bone mineral density, joint flexibility, muscle strength and balance [9]. Demura et al, found that community dwelling older adults with high scoring activities of daily living (i.e., moving from one place of the house to another, grooming and showering oneself, etc.) had lower rates of falls [10]. Mounting scientific evidence suggests that among older adult populations, remaining active through varying levels of physical activity appears protective against falls and injuries related to falls [7], [8], [11], [12].

While the risk of slips and falls is generally lower in younger adults than in older adults [13]; there may be specific subpopulations of younger/middle-aged adults employed in occupations with high slip and fall hazards (e.g., restaurant workers), where the risk of slips and falls could be as high as that observed in older adults. Verma et al. found that limited-service restaurant workers generally comprise a younger age workforce, had a high incidence of slipping [14]. They documented that specific factors in the work environment, such as floor cleaning protocols and type of worker footwear, were associated with slipping. Talbot, et al., in a qualitative study with young/middle-aged adults also noted that younger women reported a higher percentage of injuries resulting from falls when compared to any other age group [15]. Nonetheless, the relative contribution of physical activity in preventing slips and falls in younger/middle age adults is not known. In the present study, we examined the association between slips, falls and leisure-time physical activity (LTPA) among younger/middle-aged working adults (≤50 years old) working in the restaurant industry.

## Materials and Methods

This study was conducted in 36 limited-service restaurants (also known as “fast-food” restaurants or “quick-serve” restaurants where patrons generally order or select items and pay before eating and selecting their table, North American Industry Classification System Code 722211) in the states of Connecticut, Massachusetts, New York, Pennsylvania, Tennessee, and Wisconsin in the U.S. These restaurants belonged to three major chains and had similar main menu items: hamburgers and french-fried potatoes. Several approaches were used to recruit the restaurants for the study. These included approaching chains, stores or franchisees that had previously been receptive to research studies by the investigative team members, approaching restaurant trade associations, direct solicitation of stores or franchisees, and outreach via the loss control department of a large worker's compensation insurance company. Data was collected continuously throughout the two year study period.

A total of 475 workers were recruited from the 36 limited service restaurants in the years 2007 and 2008. Workers provided written consent documented on a paper-form at the time of enrollment into the study. The study and consent procedure was approved by the Institutional Review Board of the Liberty Mutual Research Institute for Safety and the Office of Human Research Administration at the Harvard School of Public Health.

### Enrollment procedure

Once permission to enroll a restaurant was received at the corporate level, members of the study team met onsite with the restaurant manager to explain the research study, obtain consent, administer a baseline manager survey, and set up an appointment to enroll and survey the restaurant's employees. Restaurant managers were given fliers advertising the study with the date of the survey team's upcoming visit to post in their employee break area. On the scheduled date, participants were enrolled, and surveys were conducted in the restaurant. Any worker employed at the specific restaurant location was eligible to participate in the study as long as they were able to understand English, Spanish or Portuguese. Those employees not working on the day of enrollment were encouraged to come to the restaurant sometime during that day, with their work shoes, if they were interested in participating in the study. The survey materials were made available in three languages: English, Spanish, and Portuguese. Enrollment procedures have been previously described in detail [14]. Participants were compensated for their time in the study in IRB-approved amounts.

### Leisure-Time Physical Activity and Demographic Characteristics

At enrollment, workers were asked to complete a baseline survey, which included their self-reported usual LTPA, height, weight, and socio-demographic characteristics (i.e., age, gender, highest educational attainment, and race/ethnicity). We assessed the worker's LTPA using a previously harmonized measure [16] that asked “How would you describe your physical activity when you are not working?” with three possible multiple choice response options:

1. Not active: mainly sedentary such as watching television, reading, or light work such as light housekeeping, light gardening or walking less than 2 hours per week;
2. Moderately active: active, light work about 2–4 hours per week; and;
3. Vigorously active: energetically, physically active more than 4 hours per week.

We categorized LTPA as: Not active, moderately active, and vigorously active during participants' leisure-time.

### Slipping

We defined a slip as a “loss of traction of the foot” [14], [17]. Following the baseline survey, workers self-reported their weekly slip experience for up to 12 weeks. Each week participants reported the number of slips and the number of hours they worked in the previous week. Participants were given a choice of reporting their weekly experience by telephone using the interactive voice response system, by an internet-based survey, or by filling out and mailing printed survey forms.

If workers experienced one or more slips, they also reported whether the slip resulted in a fall or an injury for up to four slips during the weekly survey. We operationalized a “major slip” if the participant experienced a slip that resulted in a fall and/or injury. Detailed study methods have been presented previously [14], [17]. The primary outcomes of interest were the rate of slipping (the total number of slips reported divided by total number of hours worked during follow-up) and the rate of major slipping (the total number of major slips reported divided by total number of hours worked during follow-up).

### Data Analysis

Using data from this cohort study, we restricted our analyses to young/middle-aged adults ≤50 years old (n = 433, range 18–50 years old). Participants recruited in the study are clustered within restaurants. To account for clustering of participants within restaurants, we investigated the association between the rate of slipping and the rate of major slipping and LTPA using a multivariable negative binomial generalized estimating equation model. Demographic variables that were not significant at α = 0.05 were dropped from the model. Participants over 50 years of age were excluded from the analysis. There was no missing data on physical activity or age in the age-restricted dataset used for this analysis. Missing weekly slip information was treated as missing completely at random and was not included in the analyses. All analyses were performed using SAS 9.2 statistical software package (SAS Institute, Inc, USA, 2009).

## Results

The characteristics of study participants are shown in Table 1. Approximately 42% of participants engaged in vigorous LPTA, 41% in moderate LPTA, and 17% were not physically active during their leisure time. The total number of slips reported during the follow-up was 1,127 of which 82 were classified as major slips; the total number of hours worked was 94,566 [14], [17].

**Table 1:** Socio-demographic characteristics among worker respondents (n = 433) participating in the study of Limited-Service Restaurants 2007–2008.

Characteristic	Sample Total
	n (%)†
**Age**	
< = 19 years old	115 (26.6)
20–29 years old	153 (35.3)
30–39 years old	79 (18.2)
40–50 years old	86 (19.9)
**Gender**	
Female	281 (64.9)
Male	152 (35.1)
**Education**	
Grades 0–11	152 (35.3)
High School Graduate/GED	164 (37.9)
Some College or above	116 (26.8)
**Race/Ethnicity**	
Black, Not Hispanic	90 (20.8)
Hispanic	81 (18.7)
Other	38 (8.8)
White, not Hispanic	224 (51.7)
**Primary Language**	
English	380 (87.8)
Other	53 (12.2)
**Leisure-Time Physical Activity Levels**	
Not Active	73 (16.9)
Moderate Activity	177 (40.9)
Vigorous Activity (Energetic)	181 (41.8)
**Body Mass Index** ‡	
Underweight	11 (2.7)
Healthy Weight	167 (38.6)
Overweight	112 (27.1)
Obese	123 (29.8)

† Differences in sub-total population sample due to item non-response or missing.

‡ Body Mass Index (BMI) was classified into four categories underweight (Below 18.5), Healthy Weight (18.5–24.9), Overweight (25.0–29.9), and Obese (≥30.0).

In the multivariate generalized linear model gender, BMI, job tenure, education, and ethnicity were not significantly associated with the rate of slipping and were dropped from the model. After controlling for age, the rate of worker self-reported slipping was very similar in all three categories of physical activity level (Table 2). However, the rates of major slips among workers who engaged in moderate and vigorous physical activity during their leisure-time were approximately one-third lower than the rate of major slips among inactive participants (Age-adjusted Rate Ratio (ARR) moderate LPTA  = 0.65; 95% CI [0.18–2.44] and ARR vigorous LPTA  = 0.64; 95% [0.18–2.26]).

**Table 2:** Unadjusted rate ratio and 95% confidence interval from generalized estimating equation models for self-reported slip type among workers participating in the study of Limited-Service Restaurants 2007–2008.

Slip Type	Unadjusted Rate Ratio [95%CI]	AdjustedRate Ratio (95%CI]
**All Slips** †		
Moderate Activity	0.88 [0.54–1.44]	0.81 [0.50–1.32]
Vigorous Activity (Energetic)	1.04 [0.55–1.96]	1.08 [0.60–1.94]
Age (continuous)		0.76 [0.67–0.85]
**Major Slips** †		
Moderate Activity	0.60 [0.15–2.40]	0.65 [0.18–2.44]
Vigorous Activity (Energetic)	0.63 [0.15–2.66]	0.64 [0.18–2.26]
Age (continuous)		0.70 [0.58–0.85]

† =  Reference group  =  Not Physically Active.

## Discussion

Research on the effect of LTPA on the risk of falls in younger/middle aged adults is very limited. Nonetheless, in our analyses, we did not find a statistically significant difference in the rate of major slips between workers who participated in LTPA versus those who did not. However, the observed effect estimates (Odds Ratio 0.65) were substantial. This suggests that this association should be evaluated in a larger sample of workers from which more precise effect estimates could be obtained. Our findings were not statistically significant possibly due to the relatively small number of major slips in our sample. Although these workers are actively employed in limited service restaurants where most positions involve continuous standing and frequent bouts of walking, the results observed suggest that engagement in leisure-time physical activity may still contribute as a protective factor against falling. In a case-control study on railway workers (mean age of 41 years), Gauchard reported that workers who did not participate in sporting activities were at higher odds of slips and/or falls [18]. Potential underlying mechanisms for decreased slips and falls among active younger/middle-aged adults could include improved muscle strength, balance, coordination, and reaction time as suggested by other studies [2], [9], [16].

While the well-documented risk of slips and falls is lower for younger/middle age adults when compared to older adults [16], there may be primary prevention opportunities among younger/middle-aged adults employed in high risk slip, trip and falls occupations. For example, in the present study the rate of falls among younger/middle-aged restaurant workers was 1.27 per 2,000 work hours. In contrast to this younger population, Hausdorff found that almost 40% of their participants who were 70 years and older reported falling during a 12-month follow-up period [19]. Assuming a Poisson distribution for falls and 2.5 hours of fall risk each day, the finding by Hausdroff et al. would translate to 1.07 falls per 2,000 hours- a rate lower than that of the younger restaurant workers.

This study is not without limitations. Data collected in this study are drawn from a convenience sample of restaurant agreeing to participate in the study. The sample may not adequately represent the restaurant industry, limiting the external generalizability of the study findings to other limited-service restaurants. The relatively small number of major slip incidents and the lack of objectively measured physical activity levels at both work and outside of work were the major limitations of the current study. We used a harmonized self-reported measure of LTPA that did not capture self-reported physical activity from work, an activity that could be contributing to the slip risk. Also, a more standardized self-report or objective physical activity measure for each worker may have led to less classification bias in physical activity level category. Despite these limitations, this study collected reliable and robust measures of slips in a unique high-risk occupational group.

## Conclusion

This epidemiologic study is the first to document the effects of leisure-time physical activity on slips and falls in a younger/middle-aged working adult population. Our findings were not statistically significant; however, this unique limited-service restaurant study suggests that younger/middle-aged adults who engaged in LTPA might experience less major slips than those individuals who are not physically active outside of work. The findings in this study suggest that assessment of this association in a large worker sample may be warranted. The development of primary prevention strategies in reducing slips, trips and falls in younger/middle-aged adults should consider relevant health behaviors. While our earlier work identified several modifiable work environment factors including floor friction and worker footwear, it may be possible that regular physical activity during leisure-time might offer some protection in slip and fall risk. The findings from the present study now add that leisure-time physical activity should also be further explored as a potentially modifiable factor at an individual level. Additional studies that examine the role of occupational and non-occupational physical activity on the risk of slips, trips and falls among younger and middle-aged adults, particularly in larger datasets, appear warranted.
